# Corrigendum: ChEC-seq kinetics discriminates transcription factor binding sites by DNA sequence and shape *in vivo*

**DOI:** 10.1038/ncomms15723

**Published:** 2017-06-05

**Authors:** Gabriel E. Zentner, Sivakanthan Kasinathan, Beibei Xin, Remo Rohs, Steven Henikoff

Nature Communications6: Article number:8733; DOI: 10.1038/ncomms9733 (2015); Published: 10 22 2015; Updated 05 05 2017

We have been alerted to an inconsistency regarding the control used as input in the DNA shape analysis shown in Fig. 7 of this paper.

As described in the Methods section of the Article, ‘FASTA sequences generated from the previously generated 100 bp windows centered on motif match midpoints were used as input for our DNA shape method for high-throughput prediction of DNA structural features. A set of 100 bp windows generated by BEDTools random not overlapping high-scoring or low-scoring sites and equal in number to the low-scoring sites for each factor was used as the random control for DNA shape analysis.' Thus, in Fig. 7, the transcription factor binding sites with high- and low-scoring consensus motifs were centered on the best match to the motif, while the random control sites were not.

In an accompanying Correspondence, Matthew Rossi, William Lai and B. Franklin Pugh show that when the random sites are centered on the best match to the motif, no significant difference is observed between low-scoring and random sites. We therefore cannot conclude from this analysis that the low-scoring sites identified by ChEC-seq represent shape-dependent, sequence-independent binding sites.

This inconsistency does not affect the validity of ChEC-seq as a method to identify transcription factor binding sites.

We thank Rossi, *et al*. for bringing this to our attention and direct readers to the Correspondence and our Reply, which details additional analyses of DNA shape features at ChEC-seq-identified transcription factor binding sites.

